# A Near-infrared Turn-on Fluorescent Sensor for Sensitive and Specific Detection of Albumin from Urine Samples

**DOI:** 10.3390/s20041232

**Published:** 2020-02-24

**Authors:** Yoonjeong Kim, Eunryeol Shin, Woong Jung, Mi Kyoung Kim, Youhoon Chong

**Affiliations:** 1 Department of Bioscience and Biotechnology, Bio/Molecular Informatics Center, Konkuk University, Hwayang-dong, Gwangjin-gu, Seoul 143-701, Korea; 3711992@naver.com (Y.K.); ershin94@hanmail.net (E.S.); 2 Department of Emergency Medicine, Kyung Hee University Hospital at Gangdong, Seoul 134-727, Korea; ondali77@khu.ac.kr

**Keywords:** human serum albumin, sensor, near infrared, fluorescence, urine

## Abstract

A readily synthesizable fluorescent probe **DMAT-π-CAP** was evaluated for sensitive and selective detection of human serum albumin (HSA). **DMAT-π-CAP** showed selective turn-on fluorescence at 730 nm in the presence of HSA with more than 720-fold enhancement in emission intensity ([**DMAT-π-CAP**] = 10 μM), and rapid detection of HSA was accomplished in 3 s. The fluorescence intensity of **DMAT-π-CAP** was shown to increase in HSA concentration-dependent manner (*K*_d_ = 15.4 ± 3.3 μM), and the limit of detection of **DMAT-π-CAP** was determined to be 10.9 nM (0.72 mg/L). The 1:1 stoichiometry between **DMAT-π-CAP** and HSA was determined, and the displacement assay revealed that **DMAT-π-CAP** competes with hemin for the unique binding site, which rarely accommodates drugs and endogenous compounds. Based on the HSA-selective turn-on NIR fluorescence property as well as the unique binding site, **DMAT-π-CAP** was anticipated to serve as a fluorescence sensor for quantitative detection of the HSA level in biological samples with minimized background interference. Thus, urine samples were directly analyzed by **DMAT-π-CAP** to assess albumin levels, and the results were comparable to those obtained from immunoassay. The similar sensitivity and specificity to the immunoassay along with the simple, cost-effective, and fast detection of HSA warrants practical application of the NIR fluorescent albumin sensor, **DMAT-π-CAP**, in the analysis of albumin levels in various biological environments.

## 1. Introduction

Human serum albumin (HSA, 67 kDa) is the liver-produced most abundant protein (0.6 mM) in blood plasma [1]. As HSA is synthesized in the liver, hypoalbuminemia is related with liver failure which includes cirrhosis, liver cancer, hepatitis, alcohol-related liver disease, and fatty liver disease [2]. Some people with acute heart failure [3], kidney damage [4], protein-losing nephropathy and enteropathy [5] and malnutrition [6] are also susceptible to low albumin levels. On the other hand, hyperalbuminemia, an increased concentration of albumin in blood, is caused by dehydration and high protein diets [7]. HSA also serves as “taxis” for various endogenous compounds by using several ligand binding sites located on the net negatively charged globular surface: HSA binds and transports thyroid hormones, lipid-soluble hormones, unconjugated bilirubin, free fatty acids, and divalent cations (Ca^2+^ and Mg^2+^) [8]. By the same token, many drugs bind to HSA [9], and this event has great pharmaceutical interest because, upon binding to HSA, both the pharmacokinetic and pharmacodynamic properties of the drugs are affected.

Taken together, concentration of HSA can serve as prognostic marker of morbidity and mortality [7] and a predictive factor of the pharmacokinetic properties of the drugs [10]. Therefore, development of sensitive and selective detection method for quantification of HSA in biological samples is of clinical significance. 

Various analytical methods have thus been developed for detection of HSA in blood, urine, and cell extracts, which include spectrophotometric (absorbance and fluorescence spectroscopy, and LCMS-proteomics), immunochemical (antibody), and radiochemical techniques [11,12,13]. Among those, due to high sensitivity and non-destructive nature, fluorescent measurement of HSA has attracted special addition. For this purpose, a series of molecular sensors that emit fluorescence upon binding to HSA have been discovered (Table 1).

These sensors are featured with HSA sensitivity and significant increases in the fluorescence intensities (9 ~ 400 fold) were observed upon binding to HSA (Table 1) [14,15,16,17,18,19,20,21,22,23,24]. In addition to the turn-on properties, sensors emitting fluorescent light in the near-infrared (NIR) range (650 nm–900 nm) has additional advantage of minimized interference of background fluorescence as well as deep tissue penetration. A dicyanomethylene-4H-chromene derivative (TG-SA) [14] and an indolium salt [16] represent the turn-on NIR fluorescent HSA sensors (Table 1). The fluorescent HSA sensors also exhibit favorable sensing properties including low detection limit (0.022 ~ 1.68 mg/L, Table 1) and wide detection range (Table 1). On the other hand, almost all HSA fluorescence sensors bind either to drug binding site 1 or to 2 and, due to competition with other drugs or endogenous compounds binding to the same binding site, they suffer from inaccurate determination of HSA (Table 1) [25,26,27]. In this context, sensors with the unique binding site have been expected to show enhanced HSA specificity but, to the best of our knowledge, ACDM is the sole example which targets neither drug site 1 nor 2 [15]. The accurate binding site of ACDM is still obscure, but the molecular dynamics study revealed the subdomain IB as the most probable binding site [15]. However, as ACDM emits fluorescent light at wavelength outside the NIR range (λ_em_ 612 nm), an HSA sensor with a unique binding site as well as NIR fluorescence emission has been highly anticipated to demonstrate HSA specificity with diagnostic applicability: NIR fluorescent sensors characterized by minimal background interference as well as high sample penetration depth are more appropriate to detect trace HSA in clinical samples.

Taken together, HSA sensors that have turn-on fluorescence in the NIR region with the characteristic binding site have not been disclosed, which prompted us to investigate novel scaffolds. Previously, we came to realize that a chlorinated α-cyanoacetophenone can serve as a good turn-on fluorescence sensor [28]. In this study, for fine control of HSA-selectivity as well as fluorescence emission wavelength, various aromatic moieties were incorporated into the α-cyanoacetophenone scaffold. Thus, we prepared a series of α-cyanoacetophenone derivatives conjugated with aromatic rings and, among those, (2*E*,4*E*,6*E*)-2-(3-chlorobenzoyl)-7-(5- (dimethylamino)thiophen-2-yl)hepta-2,4,6-trienenitrile (**DMAT-π-CAP**) showed HSA-selective turn-on NIR fluorescence by binding to hereto unexplored hemin-binding site.

## 2. Materials and Methods

### 2.1. Synthetic Procedure of DMAT-π-CAP (5)

Materials and reagents. Unless otherwise stated, chemicals were obtained from either Sigma-Aldrich (St.Louis, MO, USA), TCI (Tokyo, Japan), or Thermo Fisher Scientific (Waltham, MA, USA). A 400 AMX spectrometer (Bruker, Karlsruhe, Germany) (operating at 400 MHz and 100 MHz for ^1^H-NMR and ^13^C-NMR, respectively) was used to produce nuclear magnetic resonance spectra. Mass spectrometry data (*m*/*z*) were obtained by a FAB mass spectrometer (FAB-MS) at Korea Basic Science Institute (Daegu, Korea). Absorbance and fluorescence were recorded using a Cytation™ 5 system (BioTek, Winooski, VT, USA).

#### 2.1.1. Synthesis of 5-(dimethylamino) thiophene-2-carbaldehyde (**2**) 

Commercially available 5-bromothiophene-2-carboxaldehyde (**1**, 2.62 mmol), dissolved in H_2_O, was treated with dimethylamine (7.85 mmol), and the mixture was stirred at 100 ℃ for 12 h. After cooling to room temperature, water was added to the mixture, which was extracted with EtOAc three times. The organic layers were combined and washed with brine. After drying over MgSO_4_, the organic phase was concentrated under reduced pressure, and purification of the residue by column chromatography (silica gel, hexanes:acetone = 4:1) gave the desired product **2** in 92% yield as a yellow solid: ^1^H-NMR (500 MHz, acetone-d_6_) δ 9.49 (s, 1H), 7.60 (d, *J* = 4.5 Hz, 1H), 6.07 (d, *J* = 4.4 Hz, 1H), 3.11 (s, 6H).

#### 2.1.2. Synthesis of (E)-3-(5-(dimethylamino) thiophen-2-yl) acrylaldehyde (**3**)

Under N_2_, a solution of 2 (2.40 mmol) and (1,3-dioxolan-2-ylmethyl)triphenylphosphonium bromide (3.36 mmol) in dry THF was treated with sodium methoxide (6.01 mmol). After stirring under reflux for 18 h, the reaction mixture was cooled to 0 °C. The reaction mixture was treated with 2 N HCl (30 mL), stirred at room temperature for 20 min, and neutralized with 2 N NaOH. After dilution with water, the mixture was extracted with EtOAc three times. The organic layers were combined and washed with brine. After drying over MgSO_4_, the organic phase was concentrated under reduced pressure, and purification of the residue by column chromatography (silica gel, hexane:ethyl acetate:acetone = 6:1:1) gave **3** in 34% yield as a yellow solid: ^1^H-NMR (500 MHz, acetone-d_6_): δ 9.45 (d, *J* = 7.8 Hz, 1H), 7.59 (d, *J* = 15.1 Hz, 1H), 7.26 (d, *J* = 4.3 Hz, 1H), 6.00 (d, *J* = 4.3 Hz, 1H), 5.96 (dd, *J* = 7.9 Hz, 7.2 Hz, 1H), 3.09 (s, 6H).

#### 2.1.3. Synthesis of (2E, 4E)-5-(5-(dimethylamino)thiophen-2-yl)penta-2,4-dienal (**4**)

Under N_2_, a solution of 3 (0.81 mmol) and (1,3-dioxolan-2-ylmethyl)triphenylphosphonium bromide (1.14 mmol) in dry THF was treated with sodium methoxide (2.03 mmol). After stirring under reflux for 18 h, the reaction mixture was cooled to 0 °C. The reaction mixture was treated with 2 N HCl (30 mL), stirred at room temperature for 20 min, and neutralized with 2 N NaOH. After dilution with water, the mixture was extracted with EtOAc three times. The organic layers were combined and washed with brine. After drying over MgSO_4_., the organic phase was concentrated under reduced pressure, and purification of the residue by column chromatography (silica gel, hexane:ethyl acetate:acetone = 6:1:1) gave **4** in 42% yield as a red solid: ^1^H-NMR (500 MHz, acetone-d_6_): δ 9.50 (d, *J* = 10.1 Hz, 1H), 7.32 (dd, *J* = 14.0 Hz, 4.7 Hz, 1H), 7.16 (d, *J* = 18.2 Hz, 1H), 7.03 (d, *J* = 5.1 Hz, 1H), 6.43 (dd, *J* = 14.0 Hz, 4.6 Hz, 1H), 6.06 (dd, *J* = 10.1 Hz, 8.6 Hz, 1H), 5.9 (d, *J* = 5.2 Hz, 1H), 3.03 (s, 6H).

#### 2.1.4. Synthesis of (2E,4E,6E)-2-(3-chlorobenzoyl)-7-(5-(dimethylamino)thiophen-2-yl)hepta-2,4,6- trienenitrile (**5**)

Compound **4** (83 mg, 0.32 mmol) obtained above was dissolved in dry THF (5 mL), and the solution was treated with imidazole (33 mg, 0.49 mmol) and 3-chlorobenzoylacetonitrile (87 mg, 0.49 mmol) under N_2_. After stirring at 60 °C for 18 h, the reaction mixture was cooled to room temperature and concentrated under reduced pressure. Purification of the residue by column chromatography (silica gel, hexane:dichloromethane:ethyl acetate = 6:1:1) gave **5** (64.9 mg, 0.18 mmol) in 55% yield as a blue solid: ^1^H-NMR (500 MHz, CDCl_3_): δ 7.87 (d, *J* = 12.3 Hz, 1H), 7.78 (s, 1H), 7.73 (d, *J* = 6.4 Hz, 1H), 7.52 (d, *J* = 6.2 Hz, 1H), 7.42 (t, *J* = 6.8 Hz, 1H), 7.27 (s, 1H), 7.11 (t, *J* = 12.5 Hz, 1H), 7.05 (d, *J* = 12.6 Hz, 1H), 7.03 (s, 1H), 6.77 (t, *J* = 12.7 Hz, 1H), 6.41 (t, *J* = 12.8 Hz, 1H), 3.10 (s, 6H); ^13^C-NMR (125 MHz, CDCl_3_): δ 187.4, 164.2, 157.0, 153.3, 139.3, 138.9, 136.0, 134.6, 132.2, 129.7, 128.7, 126.8, 125.8, 123.0, 120.5, 117.6, 104.1, 103.8, 42.3; FAB-MS calcd for C_20_H_18_ClN_2_OS [M+H]^+^ 369.0823, found 369.0828.

### 2.2. Determination of Fluorescence Properties of DMAT-π-CAP in PBS Buffer (pH 7.4) 

Stock solutions of **DMAT-π-CAP** (500 μM in DMSO) and HSA (1 mM in PBS, pH 7.4) were prepared. At room temperature, 1 μL of HSA (1 mM in PBS, pH 7.4) and 1 μL of **DMAT-π-CAP** (500 μM in DMSO) stock solutions were mixed with 98 μL of PBS (pH 7.4). After incubation for 3 min, the mixture was transferred into 96-well plate and the fluorescence was measured (λ_ex_/λ_em_ = 600/730 nm) by a Cytation™ 5 system. All measurements were carried out in triplicates.

### 2.3. Determination of the Quantum Efficiency of Fluorescence of DMAT-π-CAP

At various concentrations (absorbances) of fluorescein (1-10 μM), integrated fluorescence intensity was measured (λ_ex_/λ_em_ = 430/515 nm) by a Cytation™ 5 system. The same experiment was performed by using various concentrations (absorbances) of **DMAT-π-CAP** (1-10 μM) in the absence and presence of HSA (10 μM). Absorbance of fluorescein (or **DMAT-π-CAP**) was then plotted against the integrated fluorescence intensity, and the resulting linear regression slope was determined to give Grad_s_ (or Grad_x_). By using fluorescein as a reference (Φ_s_ = 0.65 in water), quantum efficiency of **DMAT-π-CAP** (Φ_x_) was determined (Φ_x_ = Φ_s_[Grad_x_/Grad_s_][η_x_^2^/η_s_^2^]). As all experiments were performed in the same solvent, water, solvent refractive index of **DMAT-π-CAP** (η_x_) is equal to that of fluorescein (η_s_).

### 2.4. HSA-Selective Turn-on Fluorescence of DMAT-π-CAP

Mixtures containing 1 μL of **DMAT-π-CAP** stock solution (500 μM) and 1 μL of various bioanalytes [1 mM except BSA (200 μM)] in PBS (pH 7.4) were incubated for 3 min at room temperature. After transferring into 96-well plate, the fluorescence was measured (λ_ex_/λ_em_ = 600/730 nm) by a Cytation™ 5 system. All measurements were carried out in triplicates. The bioanalytes include HSA, BSA, Na^+^, K^+^, Ca^2+^, Mg^2+^, NH_4_^+^, PO_4_^3-^, urea, uric acid, glucose, glycine, leucine, isoleucine, lidocaine, bilirubin, γ-globulins, creatinine, pepsin, transferrin, human IgG, papain, chymotrypsinogen A, trypsin, and DNA.

### 2.5. Time-Dependent Fluorescence of DMAT-π-CAP after Incubation with HSA.

At room temperature, 1 μL of HSA (1 mM in PBS, pH 7.4) and 1 μL of **DMAT-π-CAP** (500 μM in DMSO) stock solutions were mixed with 98 μL of PBS (pH 7.4) in 96-well plate. Immediately after mixing, the fluorescence was measured (λ_ex_/λ_em_ = 600/730 nm) by a Cytation™ 5 system at every second for 3 min. All measurements were carried out in triplicates.

### 2.6. Concentration-Dependent Fluorescence of DMAT-π-CAP after Incubation with HSA

HSA stock solution (10 mM) was serially diluted with PBS (pH 7.4) to give HSA solutions with various concentrations (10, 9, 8, 7, 6, 5, 4, 3, 2, 1, 0.5, 0.25, 0.125 mM). **DMAT-π-CAP** (1 μL, 500 μM in DMSO) was mixed with 1 μL of each HSA solution in 98 μL of PBS (pH 7.4). After transferring into 96-well plate, fluorescence was measured (λ_ex_/λ_em_ = 600/730 nm) by a Cytation™ 5 system. All measurements were carried out in triplicates. 

### 2.7. Limit of Detection (LOD) of DMAT-π-CAP

In the absence of HSA, fluorescence of **DMAT-π-CAP** (5 μM, PBS pH 7.4) was measured five times, and the standard deviation of blank measurement (σ) was determined. On the other hand, in the presence of varying concentrations of HSA, fluorescence intensity of **DMAT-π-CAP** (5 μM, PBS pH 7.4) at 730 nm was measured and, from the linear plot of the fluorescence intensity against HSA concentrations, the slope was determined. Limit of detection (LOD) of **DMAT-π-CAP** was then determined by the following equation: LOD = 3σ/slope.

### 2.8. Job’s Plot Analysis of DMAT-π-CAP

Mixtures of **DMAT-π-CAP** and HSA (total concentrations = 10 μM) were generated in PBS buffer (pH = 7.4) by varying molar ratios (0.1 ~ 0.9). Fluorescence measurements were performed as described above.

### 2.9. Dissociation Constant (K_d_) of DMAT-π-CAP from HSA

Fluorescence intensity was measured (λ_ex_/λ_em_ = 600/730 nm) from incubation mixtures of **DMAT-π-CAP** (5 μM) and various concentrations of HSA (0.01, 0.02, 0.04, 0.08, 0.15, 0.3, 0.6, 1.25, 2.5, 5.0, 10, 20, 30, 40, 50, 60, 70, 80, 90, 100 μM). The titration curve thus obtained was fitted to the single site saturation binding equation [Y = *B*_max_X/(*K*_d_+X), Y: fluorescence intensity of **DMAT-π-CAP** (5 μM), X: concentration of HSA] using SigmaPlot software [23], and the dissociation constant (*K*_d_) as well as the maximum number of binding sites (*B*_max_) were then calculated.

### 2.10. Assignment of the Binding Site of DMAT-π-CAP on HSA

Stock solutions of **DMAT-π-CAP** (500 μM) and site-specific drugs (500 µM) in DMSO were prepared. Displacement of **DMAT-π-CAP** from the HSA-**DMAT-π-CAP** complex was performed by addition of one of the site-specific drugs (500 μM) including warfarin (drug binding site I, subdomain IIA), ibuprofen (drug binding site II, subdomain IIIA), and hemin (subdomain IB). At room temperature, 178 μL of albumin (10 μM) solution was mixed with **DMAT-π-CAP** (2 µL) and 20 μL of the site-specific drugs, and the resulting mixture was incubated at room temperature for 3 min. After incubation, 150 μL of the supernatant was transferred into 96-well plate and the fluorescence emission was measured (λ_ex_/λ_em_ = 600/730 nm) by s Cytation™ 5 system. All measurements were carried out in triplicates.

### 2.11. Assessment of Urinary Albumin Levels

#### 2.11.1. Fluorometric Analysis of Urinary Albumin with DMAT-π-CAP

**DMAT-π-CAP** was dissolved in DMSO to obtain a 500 μM stock solution. Urine samples were collected from healthy female donors and used at the same day after centrifugation for 10 min at 1000 *g* at 4 ℃. For direct fluorometric measurement, **DMAT-π-CAP** (5 μM) was incubated with the undiluted urine samples for 3 min, and the fluorescence emission was measured (λ_ex_/λ_em_ = 600/730 nm) by a Cytation™ 5 system. On the other hand, fluorometric standard addition method was performed by incubating **DMAT-π-CAP** (500 μM, 1 μL) and various concentration of HSA (0, 2, 4, 6, 8, 10 μM; 1 μL) in urine (98 ~ 99 μL) 3 min. The fluorescence emission was then measured (λ_ex_/λ_em_ = 600/730 nm) by a Cytation™ 5 system. All measurements were carried out in triplicates.

#### 2.11.2. Determination of Urinary Albumin by Immunoassay

For the determination of urinary albumin levels with immunoassay, human albumin ELISA kit (STA-383-Human Albumin ELISA kit Cell Biolabs, Inc., San Diego, CA, USA) was used according to the manufacturer’s protocol. The calibration curve obtained by using human HSA standard dissolved in assay buffer was used to estimate the HSA concentration in the urine sample.

### 2.12. Statistics

Data are expressed as means ± SD from at least three independent experiments. Tests used for nonparametric data included one-way analysis of variance (ANOVA) with Tukey’s post hoc test using a GraphPad Prism 5 software (San Diego, CA, USA, USA). Unless otherwise indicated, *p* values < 0.05 were considered statistically significant.

## 3. Results

### 3.1. Synthesis of DMAT-π-CAP

**Scheme 1:**
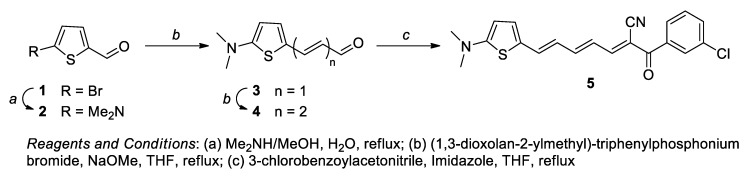
Synthesis of the title compound, **DMAT-π-CAP** (**5**).

Straightforward synthesis of **DMAT-π-CAP** (**5**, Scheme 1) was completed from commercially available 5-bromothiophene-2-carbaldehyde (**1**) in four steps. Briefly, aromatic nucleophilic substitution by dimethylamine in refluxing water converted **1** into the corresponding 5-dimethylaminothiophene-2-carbaldehyde (**2**). Chain-elongated conjugated aldehyde **3** was then prepared by aldol condensation of **2** with (1,3-dioxolan-2-ylmethyl)-triphenylphosphonium bromide followed by acidic hydrolysis. The chain-elongation reaction was repeated on **3** to give **4**, which was condensed with 3-chlorobenzoylacetonitrile under Knovenagel conditions to give the title compound **DMAT-π-CAP** (**5**).

### 3.2. Near-infrared Turn-on Fluorescence Properties of DMAT-π-CAP

**Figure 1:**
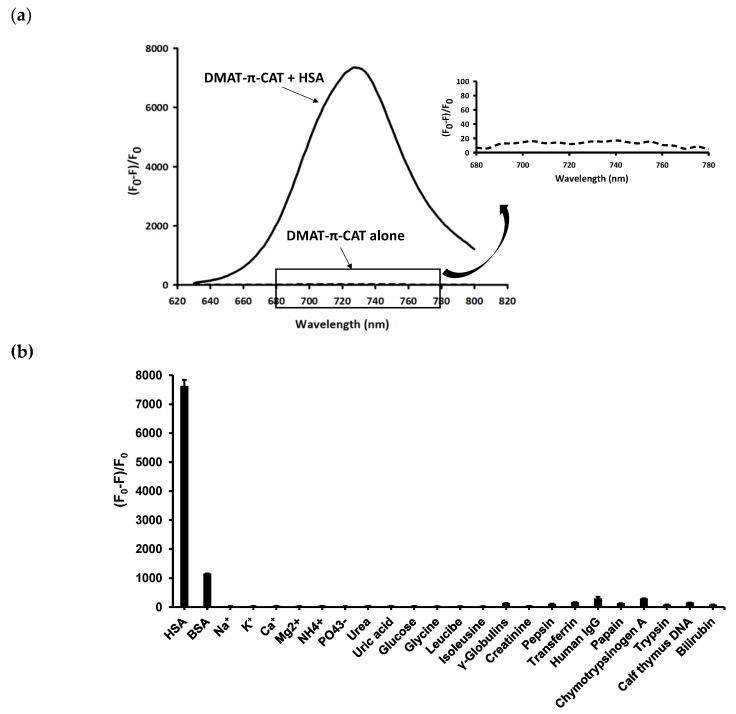
(**a**) Fluorescence spectra of **DMAT-π-CAP** (5 μM) in the absence and presence of HSA (10 μM). A magnified view of the boxed region is shown in the inset. (**b**) Fluorescence intensity of **DMAT-π-CAP** (5 μM) in the presence of various bioanalytes [10 μM except BSA (2 μM)]. (F_0_-F)/F_0_ indicates relative fluorescence intensity. Data are the mean ± standard error (n = 3).

The fluorescence properties of **DMAT-π-CAP** was investigated in PBS buffer (pH 7.4), which showed an absorption maxima (λ_ex_) at 600 nm. **DMAT-π-CAP** (5 μM) alone did not show any noticeable emission fluorescence (Figure 1a, inset) but, in the presence of HSA (10 μM, 665 mg/L), it showed strong fluorescence in the NIR region (λ_em_ = 730 nm) culminating in more than 720-fold enhancement in the emission intensity (Figure 1a) and the quantum yield increase by 37-fold (Figure S1, Supplementary Material). 

Compared with the previously reported fluorescent HSA sensors (Table 1), the remarkably high increase in fluorescence intensity of **DMAT-π-CAP** in the presence of HSA is noteworthy. Moreover, **DMAT-π-CAP** showed selective turn-on fluorescence in the presence of human (HSA) and bovine (BSA) albumin proteins, while no substantial fluorescence was observed upon co-incubation with various bioanalytes including intracellular ions, organic molecules, and proteins with wide range of isoelectric points and nucleic acid (Figure 1b). In addition, fluorescence signal from **DMAT-π-CAP** became stable in 3 s after incubation with HSA (Figure S2). Taken together, these data suggested selective and rapid binding of **DMAT-π-CAP** to HSA resulting in turn-on fluorescence in the NIR region.

**Figure 2:**
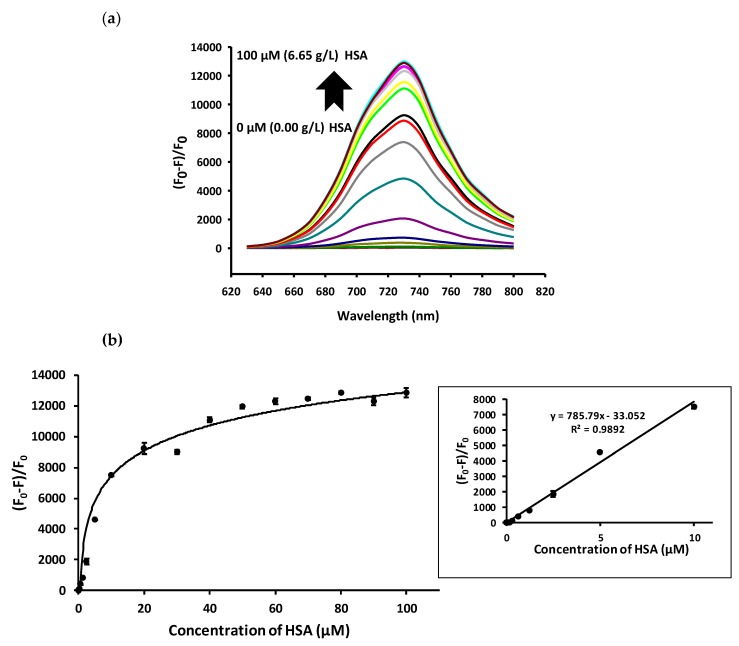
(**a**) Emission fluorescence spectra of **DMAT-π-CAP** (5 μM, λ_em_ = 730 nm) upon addition of increasing concentration of HSA (0, 1.25, 2.5, 5, 10, 20, 30, 40, 50, 60, 70, 80, 90, and 100 μM). (**b**) HSA-dependent change in the fluorescence intensity of **DMAT-π-CAP**. Inset shows the linear relationship between the fluorescence intensity of **DMAT-π-CAP** (5 μM) at 730 nm and the concentrations of HSA (0–10 μM). (F_0_-F)/F_0_ indicates relative fluorescence intensity.

The fluorescence intensity of **DMAT-π-CAP** at 730 nm was shown to increase in HSA concentration-dependent manner (Figure 2a). The steep increase in fluorescence intensity at lower HSA concentrations was followed by a plateau at 50 μM (3.3 g/L) and, at this concentration of HSA, more than 1,200-fold of fluorescence intensity increase of **DMAT-π-CAP** was achieved (Figure 2b). From the linear region of the HSA concentration-dependent fluorescence data (inset, Figure 2b; [HSA] = 0–10 μM, correlation coefficient R^2^ = 0.9892), the limit of detection (LOD) for HSA by **DMAT-π-CAP** (5 μM) was determined (LOD = 3σ/slope) to be 10.9 nM (0.72 mg/L), which is comparable to those of other HSA sensors (Table 1). 

Considering the reference range of albumin in urine (2.2–25 mg/L) [29], the wide linear dynamic range spanning two orders of magnitude (0.01–10 μM; 0.72–665 mg/L) supports the clinical use of **DMAT-π-CAP** for detecting HSA. 

### 3.3. The Binding Properties of DMAT-π-CAP to HSA

**Figure 3:**
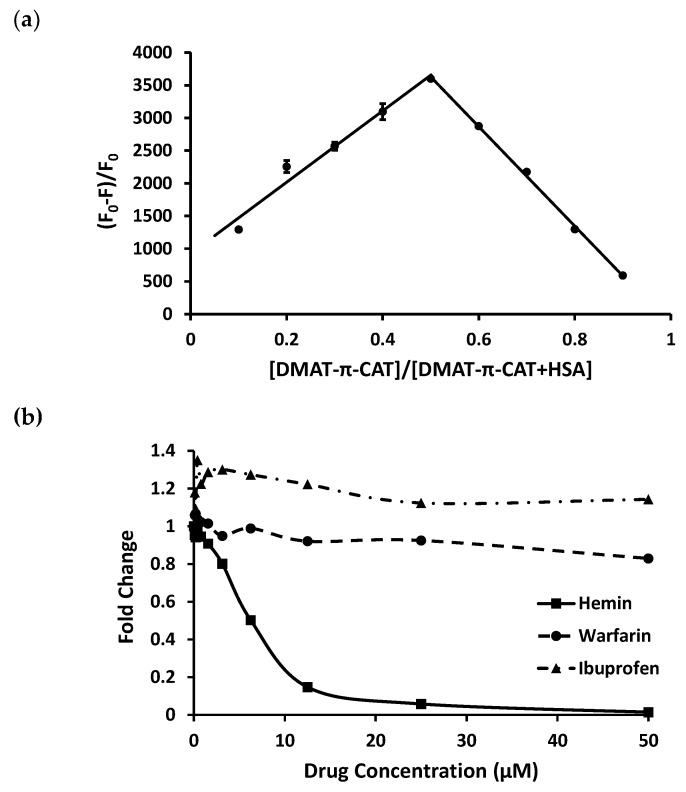
(**a**) Job’s plot analysis. Fluorescence intensity of a mixture of **DMAT-π-CAP** and HSA (total molar concentration = 10 μM at PBS) was measured at different molar ratios of the two components (0.1–0.9) (λ_em_ = 730 nm). (F_0_–F)/F_0_ indicates relative fluorescence intensity. (**b**) Changes in the fluorescence intensity (λ_em_ = 730 nm) from a mixture of **DMAT-π-CAP** (5 μM) and HSA (10 μM) upon addition of site-specific markers (warfarin, ibuprofen, and hemin).

In order to gain in-depth information of the binding properties and the binding site of **DMAT-π-CAP** on HSA, we performed Job’s plot analysis as well as competition assay with site-specific binders. First, the stoichiometry of binding of **DMAT-π-CAP** on HSA was determined to be 1:1 by analysis of a Job’s plot, which showed the maximum fluorescence intensity when the molar fraction of **DMAT-π-CAP** approached 0.5 (Figure 3a). The 1:1 stoichiometry between **DMAT-π-CAP** and HSA also suggests the single binding site and, when the one-site binding model is applied to the data shown in Figure 2b, a relatively high binding affinity with a dissociation constant (K_d_) of 15.4 ± 3.3 μM was obtained. Based on the one-site binding model, the binding site of **DMAT-π-CAP** on HSA was then explored. HSA possesses two distinct drug binding sites, and most drugs and fluorescence dyes are classified into specific binders to one of these two sites. By the same token, **DMAT-π-CAP** was anticipated to bind to either of the two drug binding sites, which could be confirmed by a competition binding assay with site-specific binders such as warfarin (drug site 1) and ibuprofen (drug site 2). However, fluorescence intensity from the preformed complex of **DMAT-π-CAP** and HSA was not affected by addition of increasing concentrations of warfarin or ibuprofen (Figure 3b). 

Instead, hemin effectively replaced **DMAT-π-CAP** from HSA to result in sharp decrease in fluorescence intensity (>95% displacement by 25 μM of hemin), which suggests that **DMAT-π-CAP** and hemin share the same binding site in HSA. Collectively, these results demonstrate that the specific binding to the hereto unexplored hemin-binding site of HSA is responsible for the turn-on fluorescence of **DMAT-π-CAP**. Hemin, a porphyrin, is a rare example of the HSA-binders targeting its subdomain IB [30]. Bilirubin [31] and lidocaine [32] are also known to bind to subdomain IB but, while bilirubin and lidocaine did not compete with **DMAT-π-CAP** for binding to HSA (Figure 1b), hemin effectively displaced **DMAT-π-CAP** from its complex with HSA (Figure 3b). This result is not surprising in light of the distinct differences in the binding sites of these subdomain IB-binding molecules (Figure S3). Thus, it might be proposed that, among the various binding sites located in the subdomain IB of HSA, only the hemin-binding site specifically accommodates **DMAT-π-CAP**.

### 3.4. Determination of Urinary HSA Levels by Using DMAT-π-CAP

**Figure 4:**
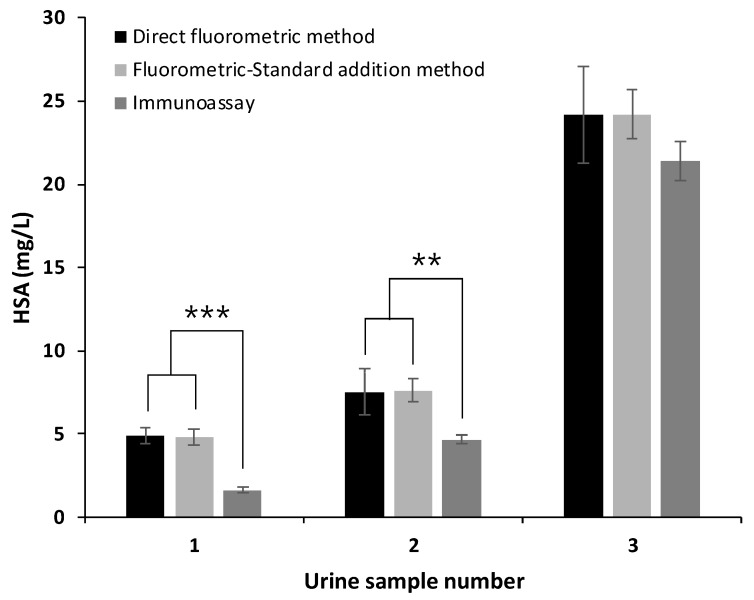
The albumin levels in urine samples determined by direct fluorometric method and standard addition method using **DMAT-π-CAP** were compared with those obtained by immunoassay. Data are mean ± SD. *, *p* < 0.05; **, *p* < 0.01, ***, *p* < 0.001.

The unique binding mode of **DMAT-π-CAP** is reminiscent of ACDM, which demonstrated selective and sensitive detection of HSA through binding to a non-drug binding site [14]. In addition, NIR fluorescence property of **DMAT-π-CAP** locates its emission wavelength at 730 nm, which is far away from the interfering background fluorescence of biological samples such as plasma (~520 nm) [33] and urine (400 ~ 450 nm) [34]. Taken together, the HSA-selective turn-on NIR fluorescence property of **DMAT-π-CAP** along with its unique HSA-binding site was anticipated to allow detection and monitoring of the HSA levels in biological samples with minimized background interference. Thus, clinical applicability of **DMAT-π-CAP** as an HSA sensor was evaluated by measurement of urinary HSA levels. Urine samples were obtained from three healthy donors, and urinary HSA levels were determined by using direct fluorometric method as well as fluorometric standard addition method. The direct fluorometric analysis is based on the proportional relationship between fluorescence emission of **DMAT-π-CAP** and concentration of HSA (Figure 2b). Therefore, fluorescence intensities of **DMAT-π-CAP** (5 μM) were measured (λ_ex_/λ_em_ = 600/730 nm) in the presence of undiluted urine samples and, by using the linear relationship between fluorescence intensity and HSA concentration (Figure 2b), the urinary HSA concentrations were determined (4.9 mg/L, 7.5 mg/L, and 24.2 mg/L) (Figure 4).

The second fluorometric measurement of urinary HSA levels was accomplished by using the fluorometric standard addition method [23], and the urine samples were spiked with various concentrations of HSA (0.2–2.0 μM; 13.3–133.0 mg/L). The fluorescence of **DMAT-π-CAP** (5 μM) in each HSA-spiked urine sample was then measured (λ_em_ = 730 nm), and the fluorescence intensity of **DMAT-π-CAP** increased linearly as the spiked HSA increased (Figure S4). From the linear plots, the urinary albumin levels of the three donors were determined to be 4.8 mg/L, 7.6 mg/L, and 22.7 mg/L, respectively, which are in good agreement with those obtained by direct fluorometric method (Figure 4). On the other hand, immunoassay using anti-HSA antibody was also performed, and the urinary albumin levels determined by immunoassay (1.6 mg/L, 4.6 mg/L, and 21.4 mg/L, respectively) (Figure S5) was comparable to those obtained by the fluorometric analyses of **DMAT-π-CAP** (Figure 4). These results demonstrate convincingly that the sensitivity and selectivity of **DMAT-π-CAP** toward HSA remains unperturbed in urine and DMAT-π-CAP can serve as a suitable fluorescence probe for precise determination of the HSA levels in urine samples.

## 4. Conclusions

In summary, a readily synthesizable fluorescent probe **DMAT-π-CAP** was evaluated for sensitive and selective detection of HSA. **DMAT-π-CAP** showed selective turn-on fluorescence at 730 nm (λ_em_) in the presence of HSA with more than 720-fold enhancement in emission intensity ([**DMAT-π-CAP**] = 10 μM), and rapid detection of HSA was accomplished in 3 s. The fluorescence intensity of **DMAT-π-CAP** was shown to increase in HSA concentration-dependent manner (*K*_d_ = 15.4 ± 3.3 μM), and the LOD of **DMAT-π-CAP** was determined to be 10.9 nM (0.72 mg/L). The 1:1 stoichiometry between **DMAT-π-CAP** and HSA was determined, and the displacement assay revealed that **DMAT-π-CAP** competes with hemin for the unique binding site, which rarely accommodates drugs and endogenous compounds. Based on the HSA-selective turn-on NIR fluorescence property as well as the unique binding site, **DMAT-π-CAP** was anticipated to serve as a fluorescence sensor for quantitative detection of the HSA level in biological samples with minimized background interference. Thus, urine samples were directly analyzed by **DMAT-π-CAP** to assess albumin levels, and the results were comparable to those obtained from immunoassay. The similar sensitivity and specificity to the immunoassay along with the simple, cost-effective, and fast detection of HSA warrants practical application of the NIR fluorescent albumin sensor, **DMAT-π-CAP**, in the analysis of albumin levels in various biological environments.

## Figures and Tables

**Table 1:**
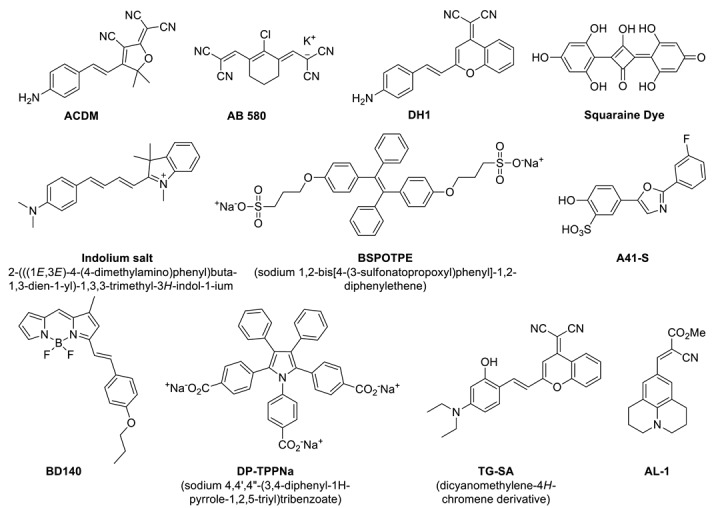
Fluorescent HSA sensors: structures, fluorescence properties, HSA sensitivity (fluorescence fold increase), binding sites and sensing properties (limit of detection, detection range).

Name	Fluorescence properties			Sensing properties (mg/L)		Binding site *^d^*	Ref
	λex (nm) *^a^*	λem (nm) *^b^*	Fold increase *^c^*	Limit of Detection	Detection range		
TG-SA	530	733	100	1.26	1.26–232	I	14
ACDM	560	612	75	2.5	0–300	ND *^e^*	15
Indolium salt	550	680	12	0.73	0.73–998	I	16
AB 580	590	616	17	0.4	1–50	NA *^f^*	17
DP-TPPNa	310	443	9	1.68	1.68–100	NA *^f^*	18
BSPOTPE	350	475	300	0.67	0–6.7	I/II *^f^*	19
A41-S	360	473	55	NA *^f^*	NA *^f^*	I	20
Squaraine Dye	560	620	80	NA *^f^*	NA *^f^*	II	21
BD140	520	585	41	NA *^f^*	NA *^f^*	II	22
AL-1	456	490	400	0.4	0–66.5	I	23
DH1	520	620	70	0.022	0-11.9	I	24

*^a^* Absorption wavelength; *^b^* Emission wavelength; *^c^* Fold increase in fluorescence intensity of the sensor after addition of HSA; *^d^* Drug binding site 1 (I) and 2 (II); *^e^* Not determined; *^f^* Not available.

## Supplementary Materials

The following are available online at https://www.mdpi.com/1424-8220/20/4/1232/s1, Figure S1: Determination of fluorescence quantum yield of DMAT-π-CAP using fluorescein as the reference, Figure S2: Time-dependent fluorescence intensity of DMAT-π-CAP (5 μM, λ_ex_ = 730 nm) bound to HSA (10 μM), Figure S3: Superimposed structures of the subdomain IB of HSA complexed with hemin (PDB ID lo9x), bilirubin (PDB ID 2vue), and lidocaine (PDB ID 3jqz), Figure S4. Fluorescence response of 5 μM DMAT-π-CAP in three different urine samples spiked with various concentrations of HSA (13.3–133.0 mg/L), Figure S5: A calibration curve for human HSA immunoassay.
