# Supplementary material for: A Near-infrared Turn-on Fluorescent Sensor for Sensitive and Specific Detection of Albumin from Urine Samples
Source: Sensors (Basel). 2020 Feb 24;20(4):1232. doi: 10.3390/s20041232 (PMC7070906; doi:10.3390/s20041232)
Supplement: Supplementary file 1 [file sensors-20-01232-s001.pdf]

A Near-infrared Turn-on Fluorescent Sensor for Sensitive and Specific Detection of Albumin from Urine Samples

Yoonjeong Kim [1,†], Eunryeol Shin [1,†], Woong Jung [2], Mi Kyoung Kim [1,*] and Youhoon Chong [1,*]

[1] Department of Bioscience and Biotechnology, Bio/Molecular Informatics Center, Konkuk University, Hwayang-dong, Gwangjin-gu, Seoul 143-701, Korea; 3711992@naver.com (Y.K.); ershin94@hanmail.net (E.S.)
[2] Department of Emergency Medicine, Kyung Hee University Hospital at Gangdong, Seoul 134-727, Korea; ondali77@khu.ac.kr
* Correspondence: mkkim@konkuk.ac.kr (M.K.K.); chongy@konkuk.ac.kr (Y.C.); Tel.: +82-2-2049-6100 (Y.C.)
† These two authors contributed equally on this work

Received: 26 January 2020; Accepted: 23 February 2020; Published: 24 February 2020

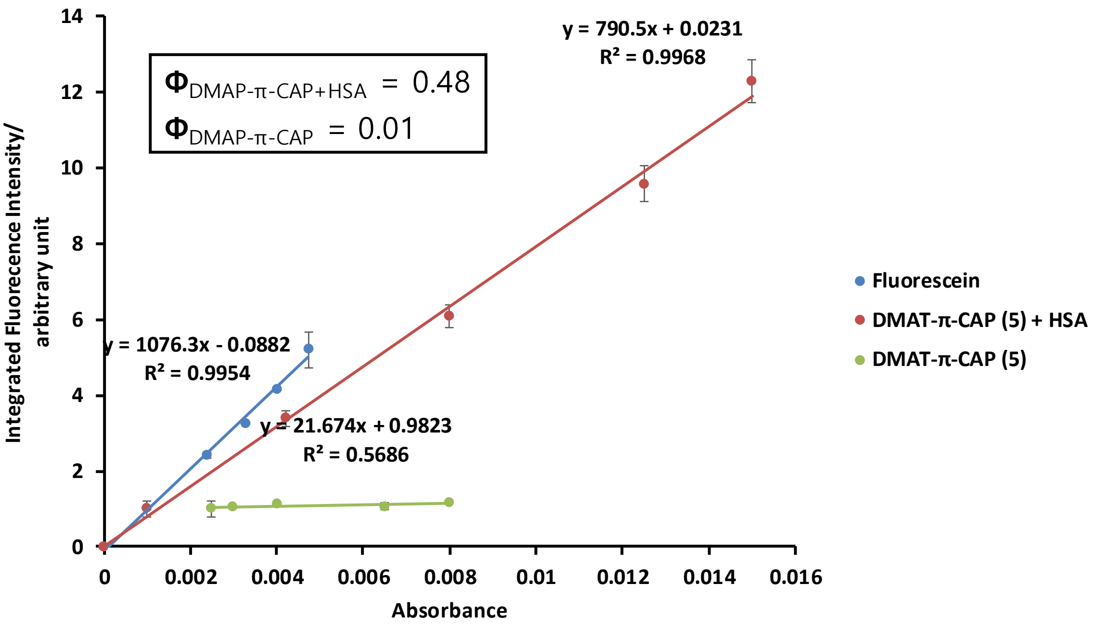

$$\Phi_X = \Phi_{ST}\left(\frac{Grad_X}{Grad_{ST}}\right)\left(\frac{\eta_X^2}{\eta_{ST}^2}\right)$$

$\Phi_{ST} = 0.65$ (fluorescein), $\eta_x = \eta_{ST}$

$Grad_{ST} = 1076.3$, $Grad_{DMAP-\pi-CAP} = 21.7$, $Grad_{DMAP-\pi-CAP+HSA} = 790.5$

$\Phi_{DMAP-\pi-CAP} = 0.013$, $\Phi_{DMAP-\pi-CAP+HSA} = 0.48$

Figure S1. Determination of fluorescence quantum yield of DMAT-π-CAP using fluorescein as the reference.

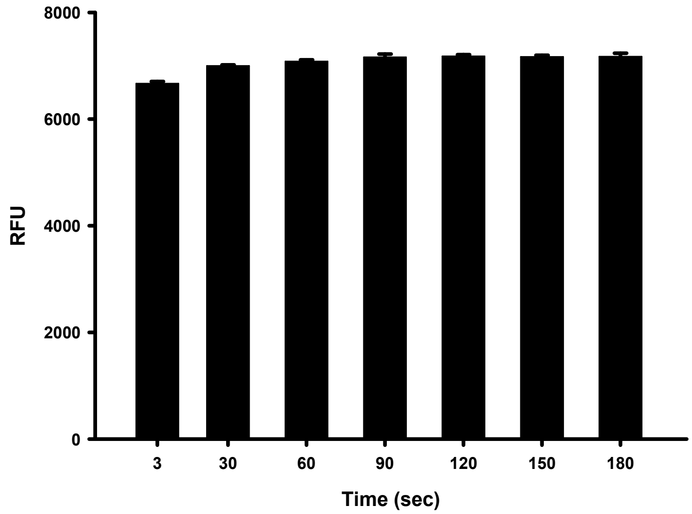

Figure S2. Time-dependent fluorescence intensity of DMAT-π-CAP (5 μM, λ_{ex} = 730 nm) bound to HSA (10 μM).

.

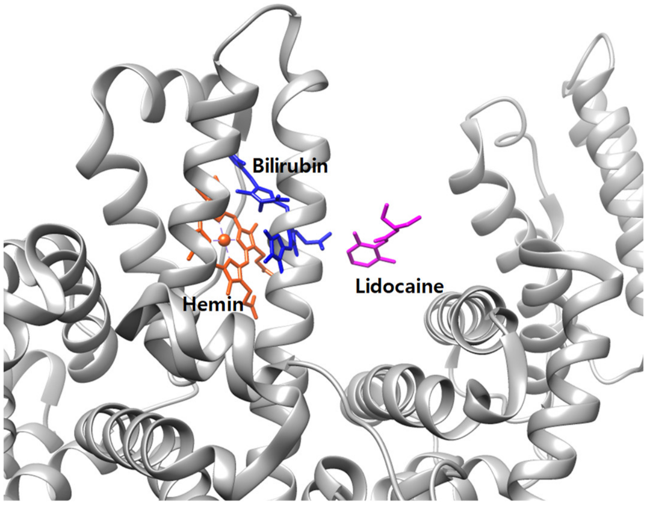

Figure S3. Superimposed structures of the subdomain IB of HSA complexed with hemin (PDB ID lo9x), bilirubin (PDB ID 2vue), and lidocaine (PDB ID 3jqz)

.

(a)

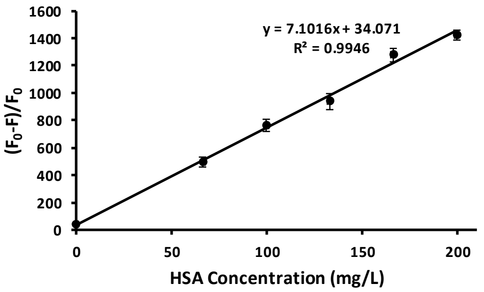

(b)

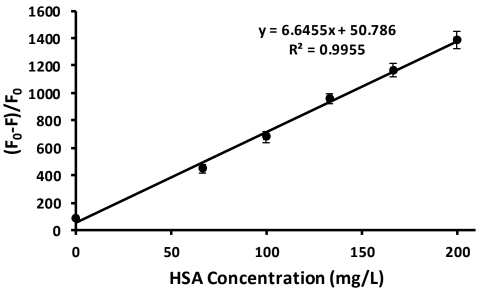

(c)

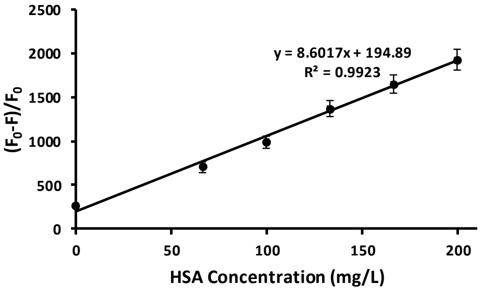

Figure S4. Fluorescence response of 5 μM DMAT-π-CAP in three different urine samples spiked with various concentrations of HSA (13.3–133.0 mg/L). (F0-F)/F0 indicates relative fluorescence intensity.

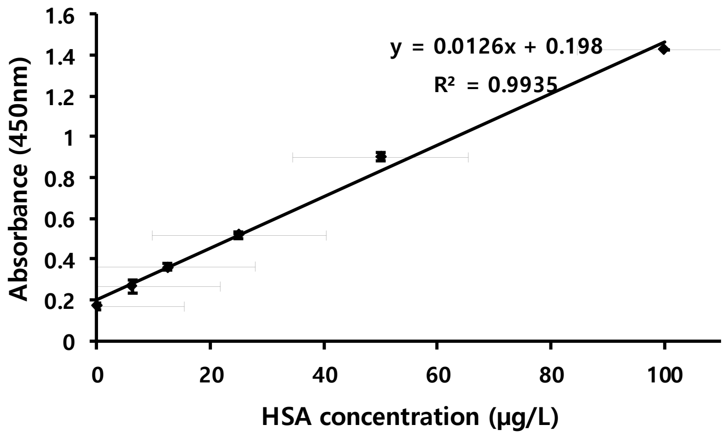

Figure 5. A calibration curve for human HSA immunoassay.
